# Atomic Layer Processes for UV-Stable Polymers: Synergistic Effects of Infiltration and Deposition of ZnO

**DOI:** 10.1021/acsami.5c14025

**Published:** 2025-11-03

**Authors:** Gil Menasherov, Nidaa S. Herzallh, Tamar Segal-Peretz

**Affiliations:** Department of Chemical Engineering, 26747 Technion − Israel Institute of Technology, Haifa 3200003, Israel

**Keywords:** atomic layer deposition, UV resistance, ZnO, optical properties, vapor phase infiltration

## Abstract

Ultraviolet (UV) radiation is the major cause of polymer degradation in outdoor environments, accelerating mechanical failure and color change, leading to plastic waste accumulation. Effective UV-protective strategies that preserve polymer functionality are therefore critical for extending material longevity in UV-intense environments. Here, we present a synergistic approach combining vapor phase infiltration (VPI) and atomic layer deposition (ALD) to engineer nanoscale zinc oxide (ZnO) coatings on poly­(lactic acid) (PLA), a UV-sensitive polymer. Individually, ALD and VPI offer minimal enhancement in UV stability; however, their sequential application enables the formation of conformal, polycrystalline ZnO films that dramatically improve UV resistance in both 3D-printed structures and thin-film PLA models. In situ microgravimetry and cross-sectional electron microscopy reveal that VPI introduces ZnO nucleation sites within and atop the polymer matrix, promoting a >10-fold increase in ZnO growth per ALD cycle. The resulting ZnO–PLA hybrids absorb over 90% of incident UV–C radiation while maintaining high optical transparency in the visible range. This low-temperature, scalable process provides a promising platform for the development of transparent, durable UV-barrier coatings on polymers for use in environmentally demanding applications.

## Graphical abstract

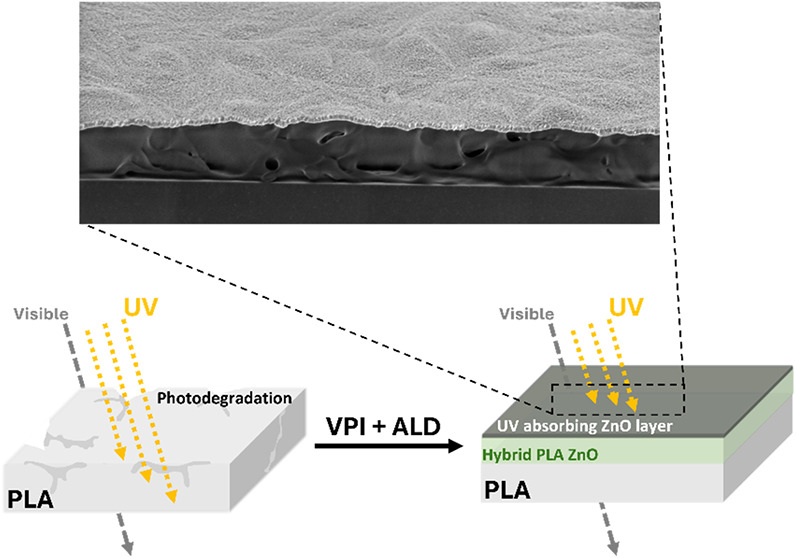

## 1 Introduction

Polymers are ubiquitous in modern society, serving as essential materials in a vast array of products ranging from personal items to industrial applications.^1^ Despite their versatility, durability, and cost-effectiveness, polymers pose a significant environmental challenge due to their slow decomposition rates.^2,3^ This slow breakdown results in the persistent accumulation of plastic and microplastics, which adversely affect marine life, agriculture, and human health.^4^ The performance and lifespan of polymer-based products are significantly impacted by environmental factors such as elevated temperatures and exposure to ultraviolet (UV) light.^5,6^ Prolonged exposure to sunlight and hence to UV radiation induces photo-oxidation, leading to chain scission and free radical formation, resulting in reduced tensile strength, toughness, and flexibility, which eventually lead to embrittlement and cracking, as well as color fading.^7^ These degradation processes shorten the products’ lifespan, necessitating frequent replacement and contributing to plastic waste accumulation.^7,8^

Current strategies for enhancing UV resistance in polymers typically involve the incorporation of UV stabilizers or additives. These may include organic compounds, such as hindered amine light stabilizers (HALS), or inorganic materials, such as zinc oxide (ZnO) and titanium dioxide (TiO_2_), which are blended with polymers during processing.^9^ An alternative common strategy is to coat the sensitive polymer with UV-resistant components such as fluoropolymers.^10^ However, since organic additives are facing increasing regulatory restrictions due to environmental concerns and inorganic additives can affect polymer properties and pose challenges in dispersion, additional solutions are needed.^9,11,12^ Recent research has demonstrated the use of techniques such as sol–gel processing,^13,14^ copolymerization,^15^ and chemical vapor deposition (CVD),^16–18^ for creating hybrid organic–inorganic and inorganic UV-protective coatings. For example, Xu et al.^18^ demonstrated that TiO_2_ coatings deposited using atmospheric-pressure plasma-enhanced CVD effectively blocked over 99% of UV light within the 200–300 nm range, while maintaining transparency to visible light. UV exposure tests on polymer films showed that the degradation rates of poly­(methyl methacrylate) PMMA and polycarbonate (PC) were reduced to one-tenth of the polymers’ degradation rates when protected with TiO_2_ coatings.^18^ Generally, sol–gel is low-cost and scales readily to large/curved parts, but it generates liquid solvent waste and can suffer nm-scale uniformity issues on textured substrates,^19^ while CVD offers high throughput at scale, but typically operates at elevated temperatures and is more line-of-sight, which can limit conformality in recessed features on heat-sensitive polymers.^20^

Within the CVD family of processes, atomic layer deposition (ALD) is a promising method for creating UV-protective coatings as it enables high precision and conformal growth of thin metal oxide layers such as ZnO and TiO_2_ on flat surfaces, as well as on high aspect ratio and complex 3D structures.^21–26^ ALD relies on the sequential exposure of a substrate to gaseous precursors that, through self-limiting adsorption and reaction, enable controlled growth of ultrathin, conformal layers with atomic-level precision. During each ALD cycle, a monolayer of precursor is adsorbed on the surface. Excess gas is then purged before the introduction of the next precursor, followed by an additional purge step. This cycle is then repeated until the desired thickness is achieved.^27,28^ Operationally, ALD is solvent-free, minimizing liquid waste but requiring reactive-precursor abatement and incurring a higher cycle cost than sol–gel. In terms of scalability, temporal (batch) ALD is size-limited, whereas spatial/atmospheric ALD enables high-throughput, large-area coating with nanometer-level uniformity.^29^ ALD has been demonstrated as an effective UV-protecting coating. For example, Xiao et al.^23^ demonstrated that nanoscale TiO_2_ coatings deposited on silk fibers via atomic layer deposition (ALD) significantly enhanced UV resistance while preserving the fiber’s structural integrity and mechanical properties.

Despite the advantages of ALD, its application to polymer substrates presents significant challenges. The primary issue arises from weak interactions between the Lewis base or nucleophilic sites on the polymer and the Lewis acid or electrophilic ALD precursors. These weak interactions result in a lack of suitable nucleation sites, which are essential for initiating ALD growth.^30–32^ This deficiency restricts ALD nucleation or requires the use of excess precursor cycles, which can result in inefficient material utilization and nonuniform film thickness.^31,33^ To address these challenges, surface activation strategies such as liquid-phase sensitization, plasma treatment, and introduction of strong Lewis base functional groups to the polymer backbone are employed.^34–36^ These methods introduce functional groups that improve the nucleation and enhance the ALD growth. However, such treatments require additional processes and may compromise the structural integrity of the polymer.

A different approach for utilizing ALD in the polymer field is through vapor phase infiltration (VPI, also named sequential infiltration synthesis, SIS), which adapts ALD principles but enables precursor diffusion into the polymer matrix. Unlike ALD, where film growth occurs at the surface, VPI involves the penetration of precursors into the polymer’s free volume, forming organic–inorganic hybrid materials.^37,38^ VPI has been successfully demonstrated in a variety of natural and synthetic polymers,^39–43^ the most prominent among them is PMMA,^44–47^ together with a growing arsenal of VPI chemistries,^44,45^ including zinc oxide (ZnO_*x*_)^48,49^ and titanium oxide (TiO_*x*_).^50^ VPI can improve polymer properties such as chemical stability,^51^ mechanical strength,^39^ and barrier performance,^52^ as well as enhance resistance to UV degradation. Azpitarte et al. demonstrated the growth of ZnO within Kevlar fiber subsurface by a VPI process and characterized its UV-protecting capabilities.^16,17^ However, VPI dispersed and, to some extent, limited growth poses a challenge for fully protecting polymers from UV-induced damage.

In this study, we explore the powerful combination of ALD and VPI to engineer nanometric ZnO coatings as UV-protective layers on UV-sensitive poly­(lactic acid) (PLA) films and 3D-printed structures. PLA was chosen as an ideal model system due to its pronounced sensitivity to UV radiation (Table S1), making it a compelling platform for testing advanced protection strategies. In addition, PLA is one of the most common materials in additive manufacturing due to its ease of processing, low melting point, and ability to form detailed and dimensionally stable structures. This enabled easy access to both thin films and macroscopic PLA 3D objects. Our findings reveal a striking contrast: while ALD and VPI alone offer minimal protection, their combination transforms the PLA surface; VPI seeds the substrate with nucleation sites that enable uniform ZnO growth during ALD, culminating in a highly effective UV-blocking layer.

## 2 Results and Discussion

### 2.1 Atomic Layer Processes of ZnO on PLA

To corroborate ZnO-ALD layer ability to protect against UV radiation, we deposited an ∼55 nm ZnO-ALD layer on a quartz substrate and measured its optical properties. The ZnO layer had high absorption between 280 and 400 nm (Figure S1), consistent with a band gap of approximately 3.37 eV and previous literature.^53,54^ In addition, the absorption of high-energy UV–C radiation (200–280 nm) by the ZnO-ALD layer is attributed to quantum confinement effects, which increase the band gap energy and shift the absorption spectrum toward shorter wavelengths.^55,56^

To probe how atomic layer processes affect the UV stability of PLA, we investigated PLA thin films that were treated with various processes, ALD, VPI, and a combined VPI+ALD process, where the VPI process is followed by ALD, as illustrated in Figure 1. ALD processes were performed in continuous mode, with exposure to DEZ and H_2_O under a constant flow of nitrogen, with 10 s intervals between pulses. In contrast, the VPI process involved extended exposure and purge times, with the system operating in static mode during the exposure step. During this step, the chamber’s inlet and outlet valves were closed, allowing the precursors to diffuse into the polymer film, leading to the growth of ZnO within the PLA film. Table 1 outlines the various process parameters for ALD and VPI, including pulse time, exposure time, and purge time for DEZ and H_2_O. All processes were performed at 80 °C.

**Figure 1:**
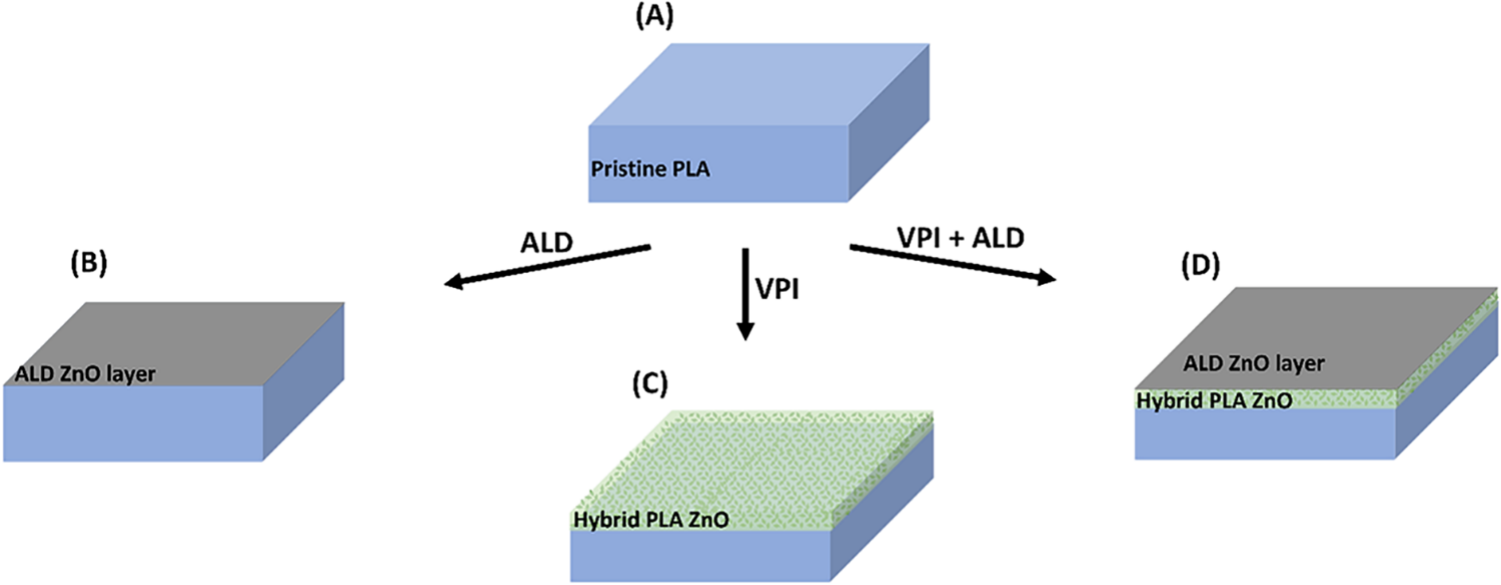
Schematic illustration of the fabrication process for a UV-protective ZnO layer using VPI and ALD. (A) pristine PLA, (B) formation of a ZnO layer on the polymer surface following an ALD process, (C) generation of a hybrid organic–inorganic interface within the polymer after the VPI process, (D) combined VPI+ALD process resulting in both a hybrid interface within the polymer and a continuous ZnO layer on the surface.

**Table 1:** ALD and VPI Process Parameters

		DEZ			H_2_O			
process	operation mode	pulse (s)	exposure (s)	purge (s)	pulse (s)	exposure (s)	purge (s)	number of cycles
ALD	continues	0.015		10	0.015		10	200–500
VPI	static	0.015	1800	900	0.015	1800	900	1–10

### 2.2 UV Resistance of the ZnO-Coated PLA Film

To quantify the ability of ZnO ALD, VPI, and the combined VPI+ALD process to enhance PLA stability under UV radiation, we exposed the ZnO-treated PLA films to UV–C radiation at a wavelength of 253.7 nm with a high intensity of 28–32 mW/cm^2^, under constant air flow, for 15 and 30 min. In this setup, the samples experience not only UV photons, but also reactive oxygen species and ozone generated under irradiation, conditions that closely resemble natural photo-oxidative environments.^57^ Upon UV exposure, PLA undergoes degradation through chain scission, breaking into smaller fragments.^58,59^ These fragments are subsequently removed by the continuous air flow, resulting in a measurable reduction in the polymer’s mass, observed as a decrease in film thickness.

Figure 2 shows the remaining thickness of the film after UV exposure, normalized to the initial film thickness prior to UV exposure. The pristine thin PLA films (∼100 nm), as well as the thin films subjected to VPI or ALD processes, showed significant photo-oxidative degradation under UV radiation, with less than ∼10% of the original thickness remaining after 30 min of UV exposure (Figure 2A). For example, PLA films treated with 10 ZnO-VPI cycles had thickness retention of only 57.7% ± 0.6 and 10.1% ± 0.9 after 15 and 30 min of UV exposure, respectively. Similarly, PLA films treated with 500 ZnO-ALD cycles showed thickness retention of 54.5% ± 0.6 and 4.9% ± 1.5 after the same exposure durations. The gradual reduction in thickness with UV exposure time indicates a progressive, surface-driven degradation, with a cumulative impact on the structural integrity of PLA thin films over time.

**Figure 2:**
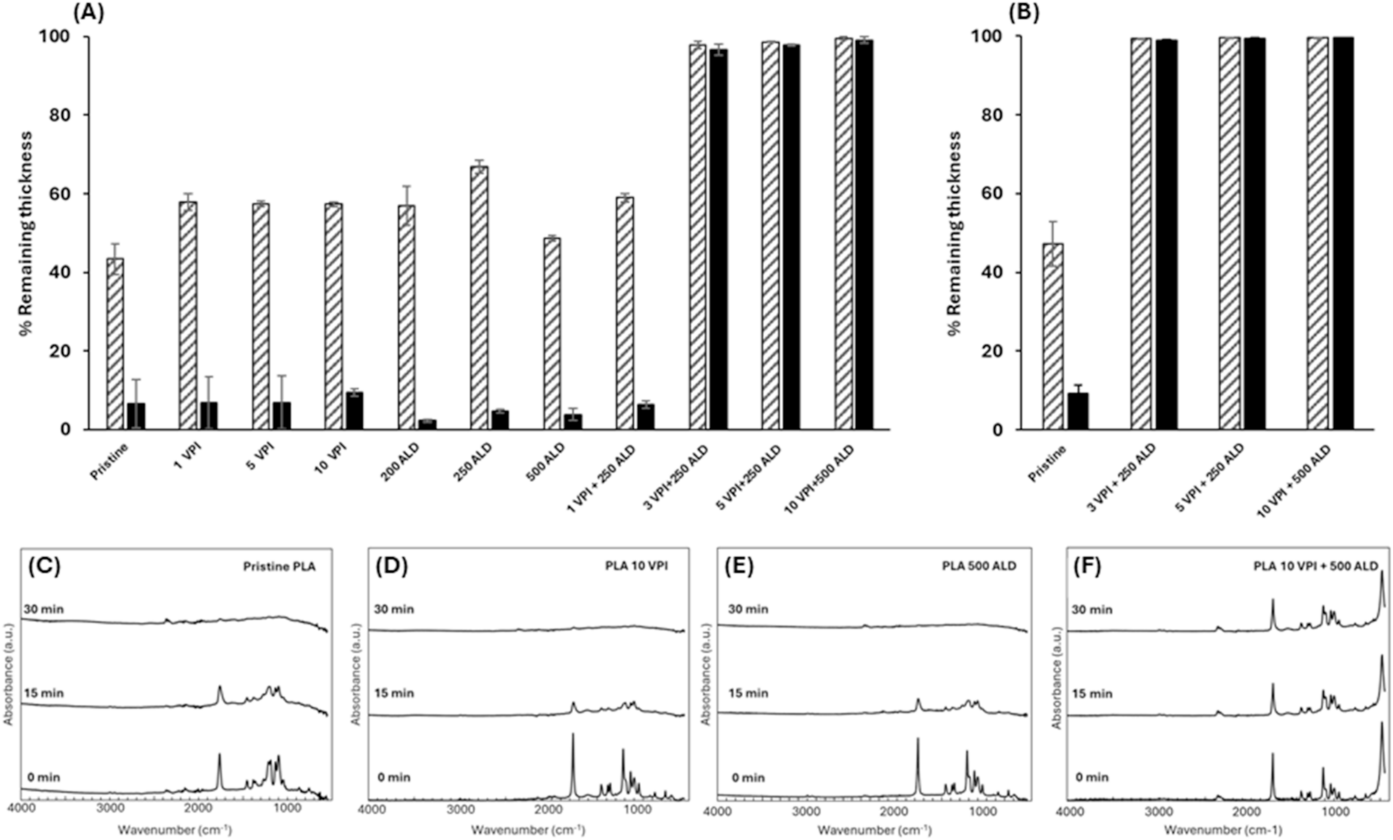
Effect of ZnO-ALD, VPI, and the combined process—VPI+ALD on PLA films’ UV resistance. (A) Percentage of remaining thickness of PLA thin films (∼100 nm) after 15 min (dashed bars) and 30 min (black bars) of UV exposure at an intensity of 28–32 mW/cm^2^ and a wavelength of 253.7 nm. (B) Percentage of the remaining thickness for PLA thick films (∼450 nm) under the same conditions. PLA films treated with ZnO-VPI+ALD retained over 96% of their thickness after 30 min of UV exposure, while pristine, ZnO-ALD, and ZnO-VPI-treated films degraded. FTIR spectra of PLA films before and after UV irradiation for 0, 15, and 30 min: (C) Pristine PLA, (D) PLA after 10 VPI cycles, (E) PLA after 500 ALD cycles, and (F) PLA after 10 VPI cycles followed by 500 ALD cycles confirmed that PLA films treated with ZnO-VPI+ALD maintained their characteristic polymer peaks after UV exposure, while pristine, ZnO-ALD, and ZnO-VPI-treated films showed significant peak reduction, indicating degradation.

Fourier transform infrared (FTIR) absorbance spectra of the PLA and ALD or VPI modified PLA films prior to and after UV exposure corroborate the polymer photo-oxidative degradation (Figure 2C–E). Pristine PLA (Figure 2C) has a prominent peak at approximately 1750 cm^–1^ that corresponds to the carbonyl (C=O) stretching vibration, indicative of the ester linkages in the polymer backbone. The C–H stretching vibrations of the methine (−CH) and methyl (−CH_3_) groups appear as absorption bands in the 2995–2945 cm^–1^ region, while the C–O–C stretching vibrations, characteristic of the ester linkages, are observed between 1180–1080 cm^–1^. Additional bands around 1455 and 1380 cm^–1^ are asymmetric bending vibrations of the methyl groups (CH_3_). Minor bands in the 1040–860 cm^–1^ range are attributed to C–C and C–O backbone vibrations. We used these spectral features to track structural changes during UV-induced degradation. For pristine PLA (Figure 2C), as well as for films subjected solely to an ALD process (Figure 2D) or VPI (Figure 2E), the polymer-related peaks described earlier are significantly reduced after 15 min of UV exposure and are completely absent after 30 min. This indicates substantial PLA degradation, suggesting that UV radiation compromises the polymer structure and leads to chain breakdown.

In contrast to films that were treated with ZnO-VPI or ZnO-ALD, films that were subjected to the combined process, with at least 3 cycles of VPI followed by at least 250 ALD cycles, demonstrated high resistance to UV radiation, retaining over 96% of the original thickness of the PLA-ZnO film, after 30 min of UV exposure (Figure 2A). For example, PLA films treated with 10 ZnO-VPI cycles followed by 500 ZnO-ALD cycles had high thickness retention of 99.6% ± 0.3 and 99.1% ± 0.7 after 15 and 30 min of UV exposure, respectively. A similar trend was observed for thicker PLA films (∼450 nm) subjected to at least 3 VPI cycles followed by ALD, where over 99% of the original thickness of the hybrid PLA-ZnO films was preserved after 30 min of UV exposure (Figure 2B). To complement thickness retention, we quantified the protective efficiency of ZnO coatings by expressing UV exposure as a UV dose (D = irradiance × time). In this study, the irradiance was ∼30 mW/cm^2^, corresponding to doses of 28 and 56 J/cm^2^ for 15 and 30 min exposures, respectively. The etch rate per UV dose was defined as $K=\frac{\left(T_{0}-T_{D}\right)}{D}$ , where (*T*_0_) is the initial thickness and (*T*_D_) is the residual thickness after UV dose D. From this metric, we defined a protection factor $\mathrm{PF}=\frac{K_{\mathrm{pristine}}}{K_{\mathrm{coated}}}$ as the ratio of the etch rate of pristine PLA (*K*_pristine_) to that of coated PLA (*K*_coated_). Pristine PLA showed high etch rates (∼2.0 nm/J cm^–2^), whereas PLA films treated with 10 ZnO-VPI cycles followed by 500 ZnO-ALD cycles showed negligible etching (∼0.02 nm/J cm^–2^). The resulting PF values for PLA thin films treated with 10 ZnO-VPI cycles followed by 500 ZnO-ALD cycles (∼125 at 28 J/cm^2^ and ∼104 at 56 J/cm^2^) demonstrate a > 100-fold reduction in degradation rate, largely independent of UV dose, confirming the robust shielding effect of the ZnO layer. Similar PF values were observed for PLA thick films treated with 10 ZnO-VPI cycles followed by 500 ZnO-ALD cycles. Interestingly, comparison of thin (∼100 nm) and thick (∼450 nm) pristine PLA films revealed markedly different degradation rates, with etch rates per UV dose of ∼2 and 8 nm/J cm^–2^, respectively. The accelerated degradation of the thicker films is attributed to their higher porosity, which facilitates deeper penetration of UV photons and reactive oxygen species (atomic oxygen and ozone) generated during irradiation. As a result, degradation proceeds not only at the surface but also throughout the film cross-section, leading to a faster mass loss. In contrast, the thinner, denser films limit photon and radical penetration, resulting in a slower, surface-dominated etching process. FTIR analysis supports these observations. For the combined process of 10 VPI cycles followed by 500 ALD cycles, the characteristic PLA peaks remain consistent across all exposure times, demonstrating effective protection of the polymer structure (Figure 2F). Additionally, a distinctive ZnO peak at 580 cm^–1^ is observed in the combined VPI+ALD-treated films, confirming the presence of the ZnO and its role in shielding the PLA from UV-induced photo-oxidative degradation. These results demonstrate that individual VPI and ALD processes provide limited improvement in PLA’s UV resistance. However, a synergistic approach, where the VPI process precedes ALD, yields a substantial enhancement in polymer resistance to UV radiation. To further quantify chemical degradation, we integrated the carbonyl (C=O) absorption band, normalized it to residual thickness, and evaluated the change per UV dose. This analysis enabled the extraction of a chemical degradation rate per UV dose and the corresponding chemical protection factor. At 56 J/cm^2^, pristine PLA exhibited a pronounced increase in normalized carbonyl intensity, consistent with extensive chain scission, whereas the PLA thin film treated with 10 ZnO-VPI cycles followed by 500 ZnO-ALD cycles showed a negligible change. The resulting chemical protection factor exceeded 320 (Table S2), indicating that the ZnO barrier suppresses photochemical bond scission by more than 2 orders of magnitude and provides stronger evidence of durability than thickness retention alone.

### 2.3 ZnO-Coated PLA Films’ Optical Properties

To evaluate the effect of ZnO on the optical properties of PLA and investigate the UV-protection mechanism, UV–vis spectra were collected for pristine PLA, PLA-ALD (500 ALD cycles), PLA-VPI (10 VPI cycles), PLA-VPI+ALD (10 VPI cycles followed by 500 ALD cycles), and a ZnO film produced by using the same combined process (Figure 3). The spectrum of the quartz substrate was subtracted from the data to isolate the effects of the deposited films. PLA films treated with ALD (PLA-ALD; black solid line) showed no significant alteration in the optical spectrum, while those subjected to the VPI process (PLA-VPI; black dotted line) exhibited minimal changes in light absorbance and transmittance in the UV range (Figure 3B, C), with no impact on the reflectance spectrum. This indicates a limited incorporation of ZnO within or on the PLA.

**Figure 3:**
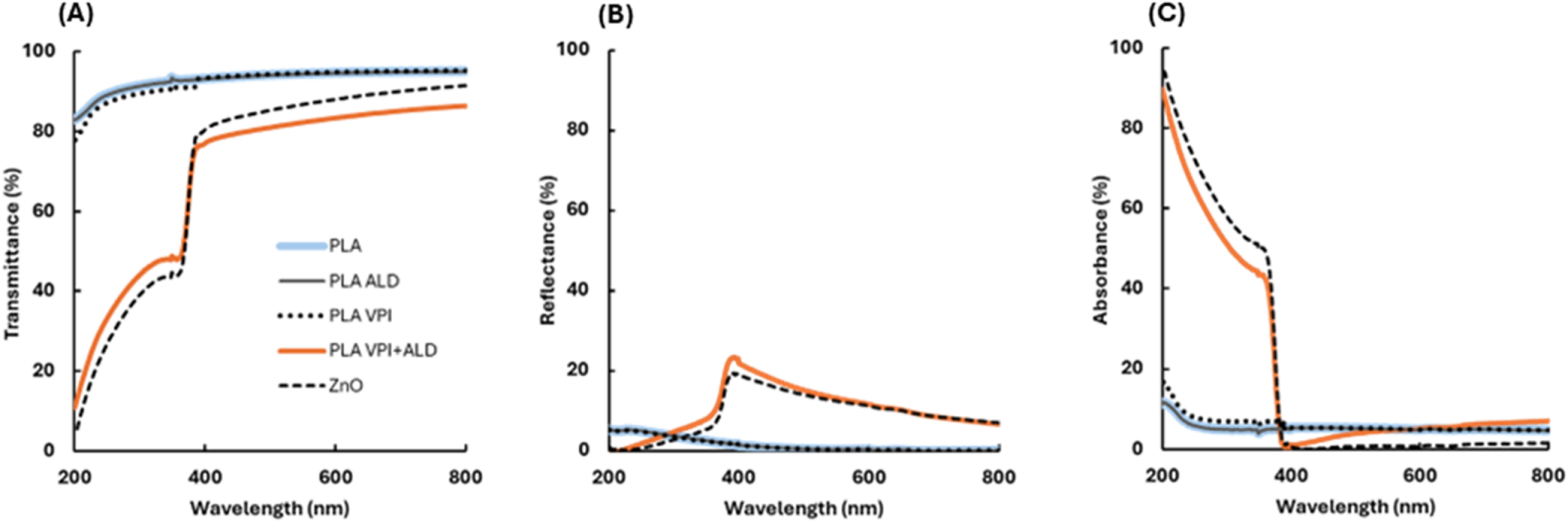
ZnO-ALD and ZnO-VPI treatments individually induce negligible changes in the optical properties of PLA, whereas the combined ZnO-VPI+ALD process significantly enhances PLA’s UV protection by increasing absorbance and reducing transmittance in the UV range, while maintaining high transparency in the visible spectrum. UV–vis spectra of ∼100 nm pristine PLA, ∼100 nm PLA film after 10 VPI, ∼100 nm PLA film after 500 ALD cycles, ∼100 nm PLA film after 10 VPI cycles followed by 500 ALD cycles, and a ∼50 nm ZnO film. (A) Transmittance %, (B) reflectance %, and (C) absorbance %.

In contrast, the combined VPI+ALD process (PLA-VPI+ALD; orange line) significantly modified the optical properties of PLA. Within the visible spectrum (400–800 nm), the ZnO layer exhibited low absorption but reflected up to 19% incident light (Figure 3B). In the UV range (200–400 nm), the ZnO layer demonstrated strong protective behavior, absorbing up to 94% of UV radiation (Figure 3C) and reflecting 0–20% depending on the wavelength (Figure 3B). For example, the transmittance at 254 nm decreased sharply from 90% for uncoated PLA to 35% after ZnO coating, indicating a robust UV protection, while at 550 nm in the visible range, transmittance decreased modestly from 95% to 83%, reflecting minimal interference with visible light transmission and confirming negligible haze, consistent with conformal ALD-grown ZnO coatings. We quantified the contributions of transmittance, reflectance, and absorbance of the various processes at two representative wavelengths: 254 nm (UV) and 550 nm (visible light). The results, presented in Figure S2, show a detailed comparison of optical behavior across these wavelengths. To further assess UV shielding, we quantified the optical blocking efficiency of the different films at 254 nm. Quantitative metrics, including optical density, effective absorption coefficient, and band-integrated blocking efficiency, are summarized in Table S3. These results highlight the large contrast between the poor UV resistance of pristine PLA and the effective UV attenuation provided by ZnO, with the combined VPI+ALD process approaching the performance of pure ZnO while maintaining high visible transparency.

The transparency of the UV-protected PLA is visually demonstrated in Figure S3, which shows a commercial transparent PLA film following treatment with 10 VPI cycles and 500 subsequent ALD cycles. The coated film displays a slight yellowish tint, characteristic of ZnO deposition, while retaining its overall optical transparency.

### 2.4 ZnO Growth within and onto PLA

To investigate the mechanism behind the observed differences in the UV protection performance among ZnO-ALD, ZnO-VPI, and the combined ZnO-VPI+ALD process, we examined the growth characteristics during the ALD stage of the growth. Figure 4A presents the average mass gain per ALD cycle, growth per cycle (GPC), measured using *in situ* microgravimetric analysis *via* quartz crystal microbalance (QCM). The GPC for the various ALD processes was determined by calculating the slope of the final 50 ALD cycles, where a steady-state growth rate was observed, as shown in Figure 4B. For conventional ALD conducted directly on the quartz crystal (250 ALD cycles), the GPC value was 74.4 ± 0.4 ng/cm^2^, consistent with previous reports on low-temperature ZnO ALD.^25^ In contrast, when the same ALD process was applied to a PLA film, the recorded GPC values were significantly lower, 5 ± 0.1 ng/cm^2^. Increasing the number of cycles to 500 had a minimal impact. This indicates minimal and nonuniform ZnO growth on PLA due to the lack of nucleation sites, attributed to weak interactions between the PLA and DEZ.

**Figure 4:**
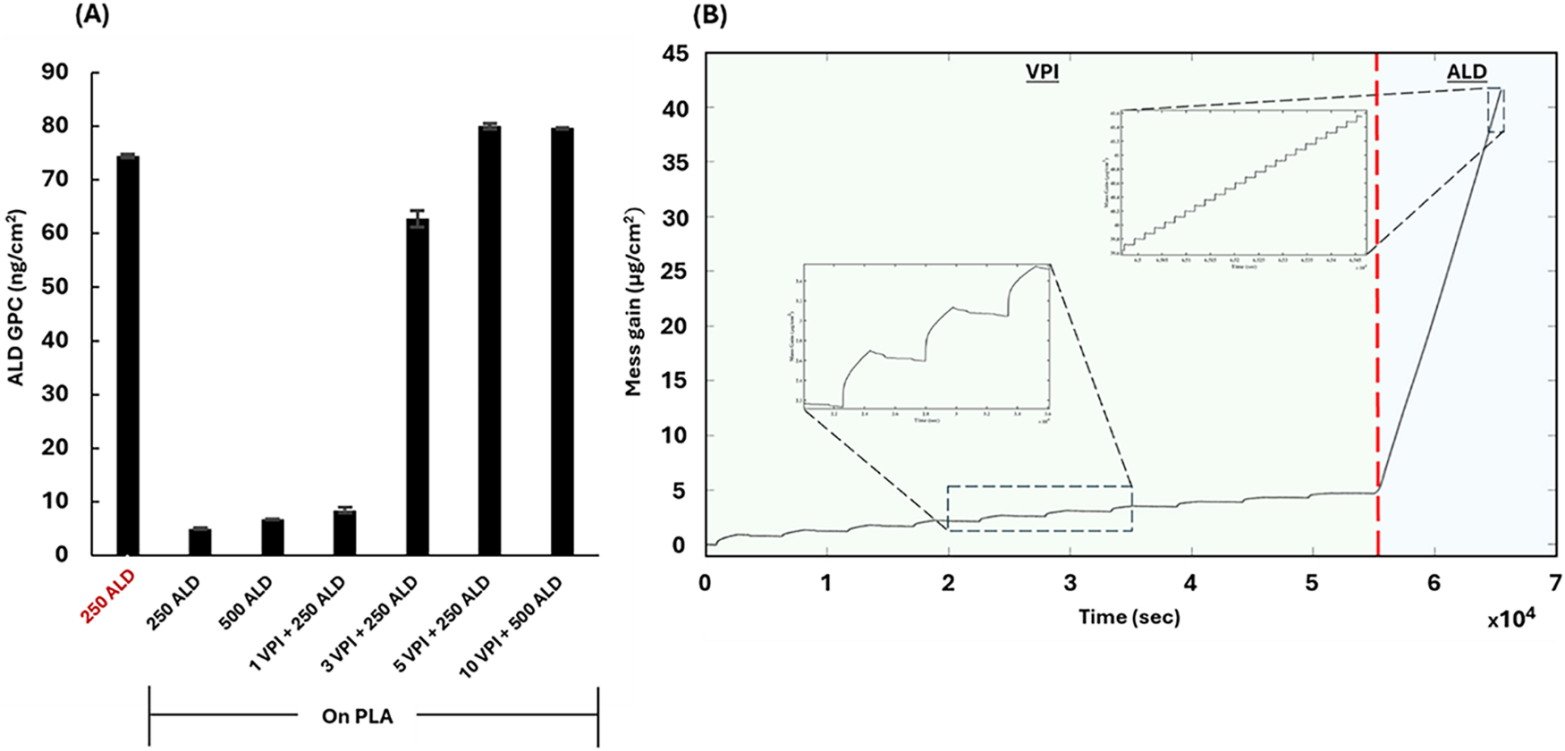
In-situ microgravimetric measurements reveal the role of ZnO VPI as an ALD-enabler on PLA. (A) ZnO-ALD average mass gain per ALD cycle (GPC, ng/cm^2^/cycle). In red, the GPC of ZnO ALD was performed on a bare quartz crystal; in black, the GPC of ZnO ALD involved various processes performed on PLA thin films. (B) QCM microgravimetric measurement during the combined process of 10 VPI + 500 ALD cycles.

Introducing VPI cycles prior to the ALD process resulted in a substantial increase in GPC values. For PLA films treated with 3 and 5 VPI cycles followed by 250 ZnO-ALD cycles and 10 VPI cycles followed by 500 ZnO-ALD cycles, the ALD GPC values were 62.8 ± 1.5 ng/cm^2^, 80.0 ± 0.5 ng/cm^2^, and 79.7 ± 0.1 ng/cm^2^, respectively (Figure 4A). An example of this VPI-promoted ALD growth can be seen in Figure 4B. First, 10 VPI cycles, with an exposure time of 1800 s, lead to an average growth of 44.4 ± 0.4 ng/cm^3^ per cycle (normalized to film thickness). Upon transition to 500 ALD cycles, a sharp change to typical ALD behavior was observed with a stable GPC of 79.7  ± 0.1 ng/cm^2^. These results indicate that the VPI cycles form nucleation sites on the PLA surface, thereby facilitating efficient ZnO-ALD growth, already from the first ALD cycles. A previous study by Weisbord et al. on ZnO VPI has shown that even 1–3 cycles can generate ZnO clusters and nanoparticles both within the polymer volume and on its surface.^49^ These clusters and particles likely serve as nucleation sites for the first ZnO-ALD cycles, allowing the rapid initiation of conformal ZnO deposition. Once a continuous ZnO layer has formed on the surface, the ZnO ALD proceeds at a constant rate. The observed increase in ALD GPC with 5 or 10 VPI cycles compared to the process where only 3 VPI cycles were used aligns with the findings of the same study, which demonstrated that the size and density of the VPI-grown ZnO nanoparticles increase with the number of VPI cycles (as illustrated in Figure S4).^49^

The difference between 3 and 5 VPI-based processes aligns with the thickness retention of thin PLA films after UV exposure (Figure 2A). After 30 min of exposure, PLA films subjected to 3 VPI cycles followed by 250 ALD cycles retained 95.7% ± 1.4 of their thickness, compared to 97.6% ± 0.3 for films subjected to 5 VPI cycles followed by 250 ALD cycles. These results suggest that additional VPI cycles promote the formation of more ZnO nucleation sites, potentially reaching a saturation state. This enhancement enables the development of a more uniform and effective protective layer during the ALD process. Notably, performing at least 3 ZnO-VPI cycles prior to ZnO-ALD leads to a GPC increase exceeding 10-fold, significantly improving UV protection performance.

To validate the role of the VPI in creating ZnO nucleation sites that facilitate efficient ZnO-ALD growth, we collected XPS for four specimens: pristine PLA, PLA after 10 VPI cycles (PLA VPI), PLA after 10 VPI cycles followed by 20 ALD cycles (PLA VPI+ALD), and PLA VPI+ALD after removal of the top ZnO layer (PLA VPI+ALD after etch). The survey spectra (Figure 5A) show that Zn features, i.e., Zn 2p doublet (∼1022/1045 eV) and Zn LMM Auger group (∼498 eV), are absent in pristine PLA, appear after the VPI process, and increase upon the subsequent ALD. The Zn features remain detectable after etching of the top (ALD) layer, indicating that Zn species are present at and beneath the polymer surface. RSF-corrected quantification (Figure 5B and Table S4) reflects the trend, with Zn atomic fractions of 0.0% (PLA), 2.7% (PLA VPI), 4.8% (PLA VPI+ALD), and 0.7% (PLA VPI+ALD after etch), consistent with the hypothesis that VPI creates nucleation sites for the subsequent ALD. The species’ chemical state was determined by combining the high-resolution Zn 2p and Zn LMM regions (Figure 5C,D) through the modified Auger parameter, α′ = BE­(Zn 2p_3/2_) + KE­(Zn LMM) which is the standard, charging-robust discriminator for Zn compounds.^60^ Using our measured values BE­(2p_3/2_) = 1022.1, 1021.9, and 1021.7 eV with KE­(LMM) = 987.6, 987.8, and 987.3 eV, we obtain α′ = 2009.7, 2009.7, and 2009.0 eV for PLA VPI, PLA VPI+ALD, and the etched sample, respectively. These α′ values fall below the typical ZnO band and indicate the formation of hydroxylated Zn (Zn­(OH)_2_) at the outermost surface, in agreement with previous reports of low-temperature, H_2_O-based growth.^61–63^ The supporting core levels, provided in the SI, are consistent with this assignment. O 1s is dominated by the PLA/–OH envelope near ∼532 and ∼533 eV and does not show a distinct ∼530 eV lattice-oxygen component expected for bulk-like ZnO, while C 1s retains the PLA ester features used for charge referencing (Figures S5 and S6). We note that the 531–532 eV band is widely recognized to include hydroxyl/adventitious contributions rather than “oxygen-vacancy” lattice signals, further supporting the hydroxylated character of near-surface zinc under our conditions.

**Figure 5:**
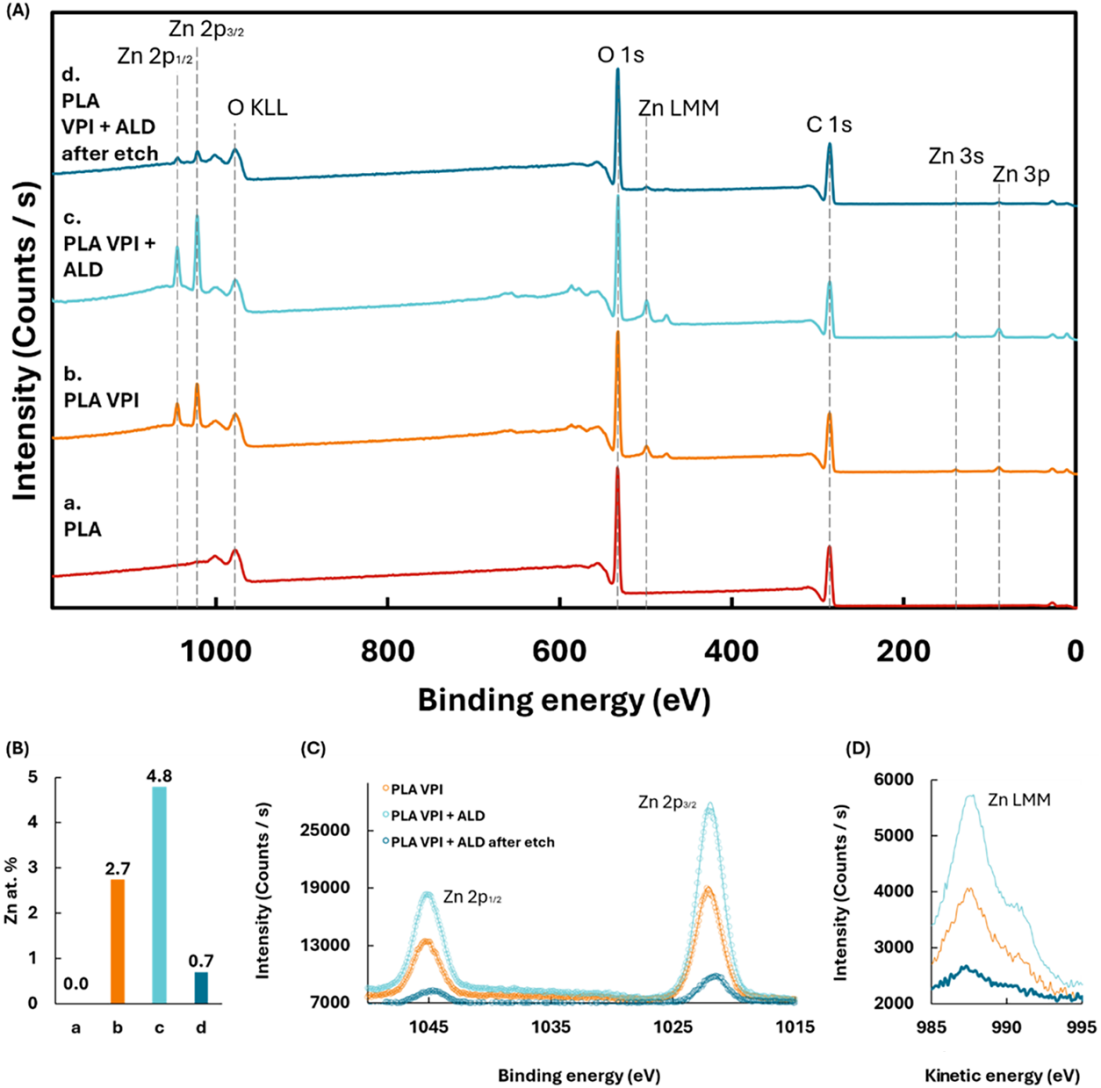
XPS shows that VPI introduces subsurface ZnO_x_ species that persist after etch and seed subsequent ALD; near-surface Zn is hydroxylated rather than bulk ZnO. (A) XPS survey spectra of (a) pristine PLA, (b) PLA after 10 VPI cycles, (c) PLA after 10 VPI + 20 ALD cycles, and (d) PLA VPI+ALD after etching of the outer layer, Zn 2p and Zn LMM emerge after VPI, increase after VPI+ALD, and remain after etch. (B) Zn atomic percentage across (a–d); nonzero Zn after etch evidence of subsurface nuclei. (C) High-resolution Zn 2p_3/2_ overlays (D) Zn LMM (kinetic-energy axis); α′ from (B, C) indicates hydroxylated Zn.

Taken together, the appearance of Zn after VPI, its persistence after removal of the outer film, and α′ values indicative of hydroxylated Zn, the XPS data support a picture in which VPI introduces subsurface ZnO_*x*_ species within PLA that act as nucleation sites for ZnO-ALD. This subsurface seeding rationalizes the immediate, high GPC observed in the subsequent ALD stage and the improved UV-protection performance of VPI+ALD coatings.^64^

To further corroborate our GPC and XPS analysis and the formation of the UV-protective layer, we characterized the films’ morphology using SEM. Figure 6 presents cross-sectional SEM images of 450 nm thick ZnO–PLA films. After 500 cycles of ZnO-ALD (Figure 6A, 6E), we observed minimal ZnO incorporation, as indicated by the weak bright signal from the ESB detector, associated with ZnO (Figure 6E). In contrast, PLA films subjected to 10 ZnO-VPI cycles (Figure 6B, F, H) showed significantly higher ZnO presence, both on the surface and within the film’s volume, as marked by the white arrow (Figure 6F, H). This observation aligns with the microgravimetric measurements, which demonstrated limited ZnO growth in the ALD process and significant growth in the VPI process.

**Figure 6:**
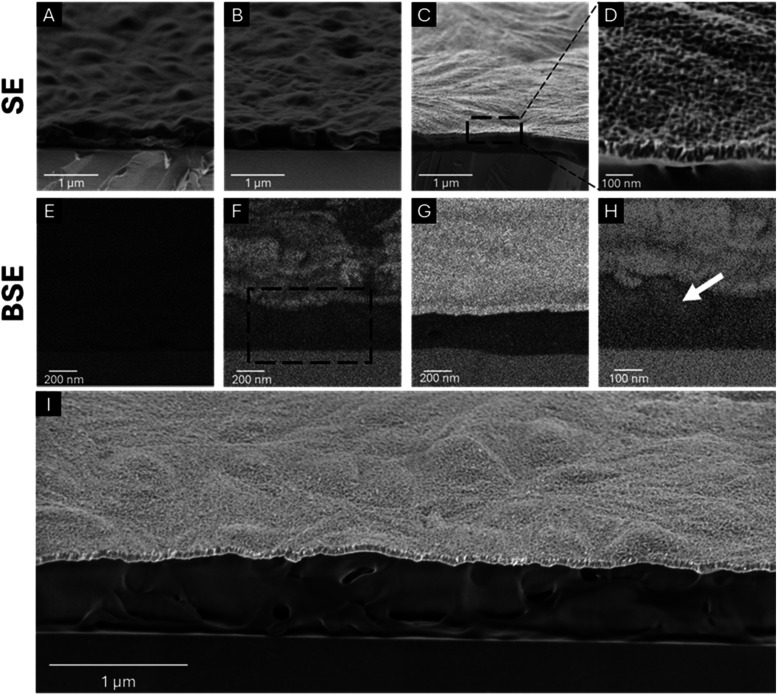
SEM cross-section images of PLA thick film (A+E) after 500 cycles of ZnO-ALD, (B+F) after 10 cycles of ZnO-VPI, and (C+G+I) after 10 ZnO-VPI cycles, followed by 500 ZnO-ALD cycles, (D) Magnification of the surface of PLA thick film after 10 cycles of ZnO-VPI and 500 cycles of ZnO-ALD, and (H) magnification of the hybrid interface of the PLA thick film after 10 cycles of ZnO-VPI.

A major difference was observed in the PLA films subjected to the combined process of 10 VPI cycles followed by 500 ALD cycles (Figure 6C, G, I). These films exhibited a high ZnO content, evident from the intense ESB signal, along with the formation of a distinct and uniform, 55 nm thick, ZnO layer on the surface (Figure 6I) and ZnO presence within the film’s volume (Figure 6G). Closer inspection and detailed analysis of the surface morphology revealed a granular surface, indicative of nanoscale ZnO growth (Figure 6D).^65^ A comparison of ZnO film thicknesses, measured by ellipsometry and presented in Table S5, shows that a ∼56 nm layer deposited via 500 ALD cycles on a Si wafer has a similar thickness as the ∼55 nm layer obtained by combining 10 VPI cycles, followed by 500 ALD cycles on a PLA film (as measured by ellipsometry, Table S5 and FIB-SEM, Figure S7). This similarity in ZnO thickness supports the proposed ALD growth mechanism, wherein the VPI pretreatment plays a minor role in UV protection; however, it promotes effective ZnO nucleation and uniform film formation on the polymeric substrate. The resulting ZnO layer plays a critical role in enhancing the UV stability of PLA by absorbing incident UV radiation and preventing its penetration into the polymer, thereby mitigating UV-induced degradation.

To further investigate the structural characteristics of the hybrid films, we analyzed the nanostructure of the ZnO–PLA films using high-resolution transmission electron microscopy (HR-TEM, Figure 7A) and energy-dispersive X-ray spectroscopy (EDS, Figure 7B–D). To achieve this, thick (250 μm) commercial PLA films underwent the combined process of 10 VPI cycles followed by 500 ALD cycles and were subsequently cross-sectioned using focused ion beam (FIB) milling. HR-TEM imaging provided direct evidence of the polycrystalline nature of the ZnO layer formed on the PLA surface, while EDS mapping revealed ZnO infiltration (a few nanometers) into the polymer–surface interface. These findings further validate the microgravimetric and SEM analyses, demonstrating that the VPI process plays a crucial role in facilitating ZnO incorporation within the polymer matrix. The observed nanoscale infiltration and uniform surface deposition of ZnO confirm the formation of a well-integrated hybrid structure.

**Figure 7:**
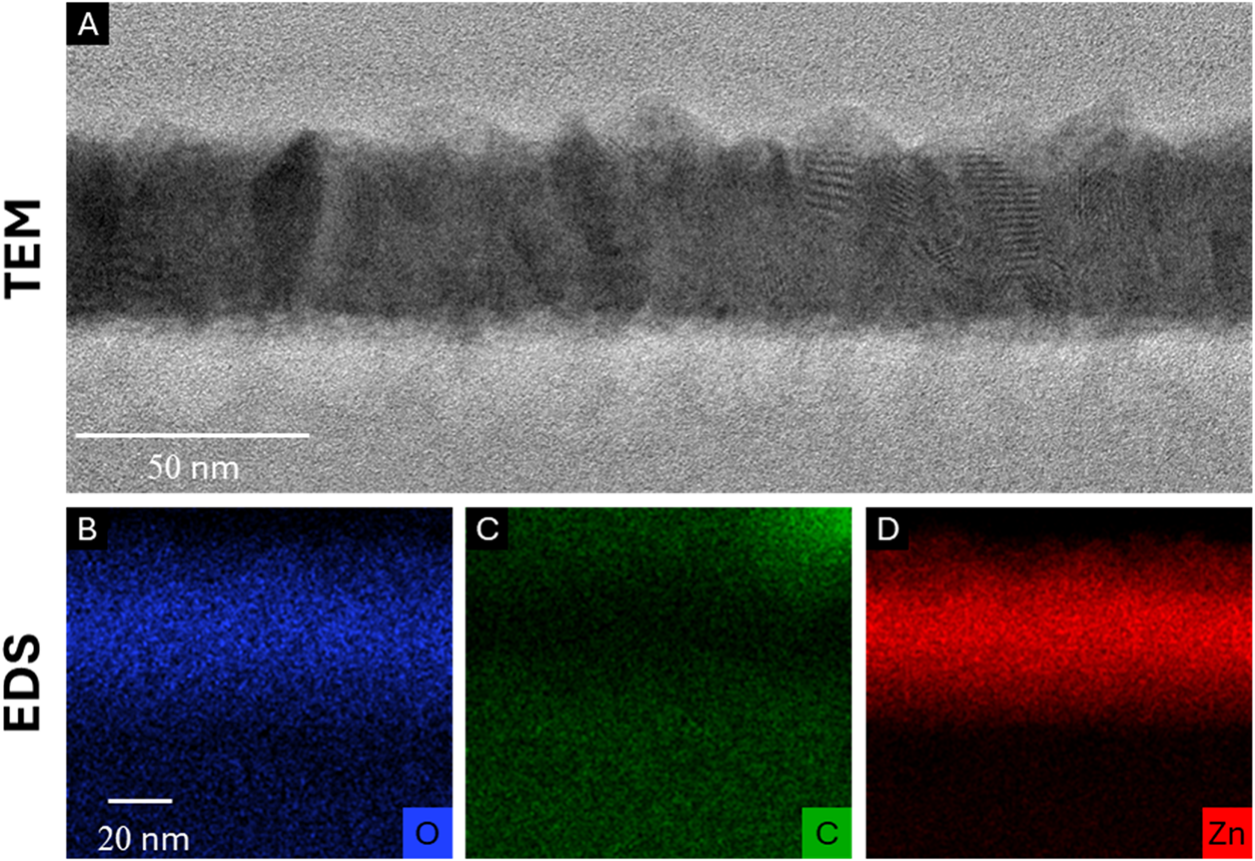
High-resolution transmission electron microscopy (HR-TEM) and energy-dispersive X-ray spectroscopy (EDS) analysis of a thick (250 μm) ZnO–PLA hybrid film. (A) HR-TEM image showing the polycrystalline structure of the ZnO layer formed on the PLA surface. (B–D) EDS elemental mapping of the cross-sectioned ZnO–PLA film, highlighting ZnO infiltration a few nanometers into the polymer–surface interface.

### 2.5 UV Resistance of ZnO-Coated, 3D-Printed PLA Mesh Structures

Finally, to demonstrate the effectiveness of ZnO coating via the combined VPI+ALD process for UV protection at the macroscale, we 3D-printed PLA mesh structures (2.8 cm × 3.6 cm) as representative models of macroscopic three-dimensional polymer architectures. We then exposed the meshes to prolonged UV–C radiation at a wavelength of 253.7 nm with an intensity of 2.5 mW/cm^2^, for 7 consecutive days. This exposure simulated approximately 220 days of UV exposure at sea level during solar noon. After UV exposure, the pristine PLA meshes became extremely brittle and broke into small fragments upon contact (Figure 8A). Employing ZnO-ALD processes or ZnO-VPI processes did not improve the PLA mesh stability to UV radiation. In contrast, the PLA meshes treated with the combined ZnO-VPI+ALD process (10 VPI cycles followed by 500 ALD cycles) stayed completely intact (Figure 8B). Moreover, the treated meshes also retained their flexibility, as shown in Figure 8C, D. These proof-of-concept 3D models demonstrate the ability to combine ZnO-VPI and ALD processes for protecting PLA structures from UV radiation while preserving their mechanical properties.

**Figure 8:**
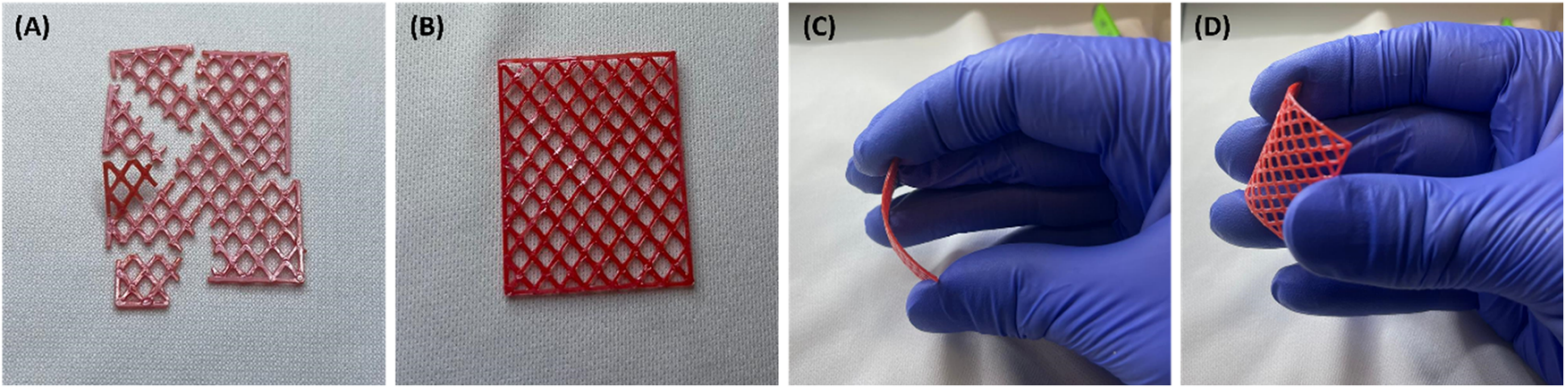
ZnO-VPI+ALD treatment significantly enhances the UV stability of 3D-printed PLA mesh structures, preventing fragmentation and preserving flexibility. (A) Pristine PLA mesh after exposure to 7 days of UV–C radiation (253.7 nm, 2.5 mW/cm^2^), simulating approximately 220 days of solar exposure at sea level. The untreated PLA became extremely brittle and broke into small fragments upon contact. (B) PLA mesh treated with the ZnO-VPI+ALD process (10 VPI cycles followed by 500 ALD cycles) remained intact after identical UV exposure, demonstrating improved stability. (C, D) The treated PLA meshes retained their flexibility, in contrast to the structural degradation observed in the untreated sample, highlighting the protective effect of the ZnO-VPI+ALD coating in maintaining the mechanical integrity of the polymer.

## 3 Conclusions

This study demonstrates a novel approach to enhancing the UV resistance of PLA by integrating vapor phase infiltration (VPI) and atomic layer deposition (ALD) processes to create ZnO coatings. Our results show that while stand-alone VPI or ALD processes did not yield polymer protection to UV, the combined VPI and ALD significantly enhanced the UV durability of PLA films and 3D-printed structures, preventing photo-oxidative degradation under intense UV exposure. The hybrid PLA-ZnO films with at least 3 VPI cycles, followed by ALD, exhibited excellent resistance to UV radiation, retaining over 96% of their original thickness after UV exposure. This protective effect is attributed to the ZnO layer’s ability to absorb and convert harmful UV radiation into harmless infrared radiation, preserving the polymer’s structural integrity.

Microgravimetric analysis using QCM confirmed the ability of ZnO-VPI to enhance ZnO-ALD growth on PLA films. A 10-fold increase in the ZnO-ALD GPC was observed when at least three VPI cycles preceded the ALD process, enhancing the uniformity and density of the ZnO coating. Electron microscopy analysis corroborated these results, confirming the robust ZnO formation on and within the PLA films. This study demonstrates the potential of integrating VPI and ALD processes to develop durable, UV-resistant coatings that could extend the lifespan of polymer-based materials in UV-intensive environments.

## 4 Experimental Section

### 4.1 Materials

The polymer substrates used in this study were obtained by spin-coating PLA onto a silicon wafer (thickness 280 ± 20 μm, orientation P ⟨100⟩), a Au-coated silicon wafer (1500 Å of Au), and a quartz crystal (thickness 1 mm, fused). The polymers spin-coated on silicon wafers were used for UV exposure testing, thickness measurements, and scanning electron microscopy (SEM) measurements, while the polymers spin-coated on Au-coated silicon were used for Fourier transform infrared (FTIR) analysis, and quartz crystal wafers were used for in situ microbalance microgravimetric measurements. PLA powder was purchased from Sigma-Aldrich. PLA filaments were purchased from ESUN, and commercial PLA film was obtained from NatureWorks for research purposes. Chloroform (99%), which was used as the solvent for the spin-coating of PLA, was purchased from Sigma-Aldrich and used without further purification. The diethyl zinc precursor for coating ZnO was purchased from Sigma-Aldrich.

### 4.2 Preparation of PLA Films and 3D Mesh

The Au-coated QCM crystal was spin-coated with PLA and stabilized for 1 h at 100 °C in a nitrogen environment prior to the VPI or ALD processes. The polymeric films were also spin-cast on the Si (1 0 0) substrates (resistivity <0.005 Ω·cm) with a native oxide layer of ∼1.5 nm. The thickness of the polymer films was measured using an ellipsometer (α-SE, J. A. Woollam Co. Inc.), whose data were fitted with a stack film model composed of a Cauchy model for the polymer layer and a Si model for the substrate.

3D-printed structures were printed using a red PLA filament. PLA structures were printed in an enclosure using a 60 °C heated bed temperature and 200 °C nozzle temperature.

ALD and VPI experiments were conducted in a commercial ALD system (Savannah S100, Veeco). For ZnO formation, the vacuum phase DEZ and H_2_O are used as reactants. To achieve thermal equilibrium and remove excess volatile species, all of the samples were subjected to 50 standard cubic centimeters per minute (sccm) of N_2_ flow at 0.3 Torr for at least 30 min. All reactants were introduced to the reactor by a 5 sccm carrier gas (99.999% N_2_). Pulsing time of each precursor was fixed at 0.015 s. Both VPI and ALD of the films and 3D-printed models were carried out at 80 °C.

The parameters for both the ALD and VPI processes remained consistent throughout all experiments, with the only variable being the number of deposition cycles. The Au-coated QCM crystal was spin-coated with a PLA film and stabilized for at least 2 h prior to ALD or VPI processes. A full ALD cycle consists of pulse (DEZ, 0.015 s) → purge (N_2_, 10 s) → pulse (H_2_O, 0.015 s) → purge (N_2_, 10 s). By contrast, the infiltrated models were prepared by VPI. In the VPI case, the substrate was exposed to the precursors for defined periods of time before purging, thereby allowing diffusion of the precursors into the polymer. A VPI cycle consisted of pulse (DEZ, 0.015 s) → exposure (1800 s) → purge (N_2_, 900 s) → pulse (H_2_O, 0.015 s) → exposure (1800 s) → purge (N_2_, 900 s).

### 4.3 Characterization of Coated Polymers

**Scanning electron microscopy** (SEM, ZEISS Ultra+) was used to determine the morphologies of the ZnO-coated polymers and the thickness of the hybrid layer. **Fourier transform infrared (FTIR) spectroscopy** was used to confirm the chemical structure of the polymers and ZnO-coated polymers before and after UV exposure. The stabilities of polymers with and without ZnO coatings against UV irradiation were measured using the changes in the FTIR absorbances and changes in film thickness using the α-SE Spectroscopic Ellipsometer—Cauchy model. PLA thin films were subjected to **UV irradiation** from a UVO Cleaner model 18 (wavelength of 253.7 nm with an intensity of 28–32 mW/cm^2^). The UVO Cleaner model 18 was purchased from Jelight Company, Inc. The samples were placed inside the UVO Cleaner model 18 at a distance of 5 mm from the UV source. PLA 3D models were exposed to UV radiation from a Spectrolinker XL-1000, at a wavelength of 253.7 nm (UV–C) with an intensity of 2.5 mW/cm^2^. **UV–vis transmission and reflectance spectra** were measured using a UV–vis–NIR spectrophotometer (Cary 5000; Agilent Technologies, Santa Clara, CA) with the use of an internal diffuse reflectance accessory (DRA) optical integrating sphere (Agilent Technologies, Santa Clara, CA). **X-ray Photoelectron Spectroscopy (XPS)** spectra were acquired with Al Kα radiation (1486.6 eV) using a 650 μm analysis spot. Survey scans were recorded at 200 eV pass energy; high-resolution regions (C 1s, O 1s, Zn 2p, Zn LMM) were collected at 20 eV pass energy (Zn LMM is shown on a kinetic-energy axis). For the ALD specimen, measurements were taken before and after etching of the outer layer by argon-gas cluster sputtering (Ar_75_^+^, 8 kV, 60 s, 2 mm raster). **Cross-sectional transmission electron microscopy (TEM)** was used to study the microstructure of the ZnO layer. For TEM imaging, an ∼55 nm ZnO layer was applied directly on a commercial PLA film with a thickness of 250 μm. The film was coated with a 10 nm conductive layer of iridium to reduce electron charging (Compact Coating Unit 010, Safematic). **Plasma-focused ion beam milling** (Helios 5 PFIB CXe Dual-Beam, Thermo Fisher) was used to prepare cross-sectional lamellae. Prior to the milling, platinum and carbon coatings (∼15 and 125 nm, respectively) were deposited onto the top of the iridium layer to protect the underlying layers. Images were taken using a high-resolution transmission electron microscope (Titan Themis G2 60–300 FEI, Thermo Fisher) at 60 keV, with bright-field TEM and high-angle annular dark-field (HAADF) scanning TEM (STEM). Energy-dispersive X-ray spectroscopy (EDS) STEM with a dual EDS detector was used for elemental mapping.

## Supplementary Material



## Notes

ALD processes data, UV–vis measurements, and optical properties, XPS, FIB-SEM (PDF)
