# Supplementary material for: Atomic Layer Processes for UV-Stable Polymers: Synergistic Effects of Infiltration and Deposition of ZnO
Source: ACS Appl Mater Interfaces. 2025 Nov 3;17(51):69885–96. doi: 10.1021/acsami.5c14025 (PMC12794801; doi:10.1021/acsami.5c14025)
Supplement: Supplementary file 1 [file am5c14025_si_001.pdf]

Supporting Information

Atomic layer processes for UV-stable polymers: synergistic effects of infiltration and deposition of ZnO

Gil Menasherov[1], Nidaa Herzallh[1], Tamar Segal-Peretz[1*]

[1] Department of Chemical Engineering, Technion – Israel Institute of Technology, Haifa 3200003, Israel

*Corresponding author, Email: tamarps@technion.ac.il

1. Polymers UV sensitivity

The sensitivity of polymers to UV radiation is intrinsically linked to their molecular structure, as demonstrated by the wide variation in UV stability among different polymers (Table S1). Polymers such as polyethylene and polypropylene exhibit poor UV stability, which is reflected in significant changes in their tensile properties and hardness upon UV exposure. This susceptibility is primarily due to their simple molecular architectures, composed of linear or lightly branched chains that are prone to UV-induced chain scission.[1–4] Conversely, polymers with more complex structures such as poly(methyl methacrylate) (PMMA) exhibit better UV stability. The inclusion of methyl groups in PMMA's molecular structure enhances resistance to UV degradation, thereby preserving its molecular integrity even under prolonged UV exposure.[1,3,5] Additional factors affecting UV sensitivity include crosslinking, which enhances structural integrity by limiting the spread of chain scission, and crystallinity, where tightly packed regions resist photochemical reactions while amorphous regions are more susceptible to UV-induced breakdown. Functional groups, particularly unsaturated bonds and carbonyls, also contribute to vulnerability by absorbing UV light and forming reactive radicals that accelerate degradation.[6,7]

Polylactic acid (PLA) stands out as a particularly vulnerable material among the polymers with varying sensitivities to UV radiation, as highlighted in Table S1, making it an ideal model system for investigating strategies to enhance UV resistance. Moreover, PLA is one of the most common materials in additive manufacturing due to its ease of processing, low melting point, and ability to form detailed and dimensionally stable structures.

Table S1. Comparison of polymers' UV sensitivity and stability characteristics.

Polymer	Chemical structure	UV sensitivity range nm	UV stability	Main UV effects	Ref.
Polyethylene	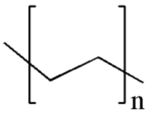	260-360	Poor	Change in tensile properties and hardness Decrease in average molecular weight	1,8
Polypropylene	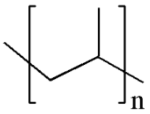	315-330	Very poor	Change in tensile properties and hardness	1,8
Poly (ethylene terephthalate)	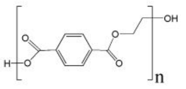	280-320	Good	Product yield	1,8
Poly(methyl methacrylate)	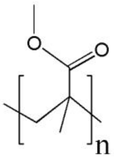	300	Very good	Decrease in average molecular weigh	1,8
Polylactic acid	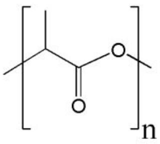	200-400	Very poor	Change in tensile properties and hardness Discoloration Initiation of additional degradation processes	9,10

2. Quantification of UV resistance of ZnO-coated PLA film

We quantified photo-oxidative damage by tracking the growth of the carbonyl (C=O) absorption band and normalizing it to the residual film thickness at each UV dose. The normalized carbonyl area is defined as: $A(D) = \frac{\int_{\upsilon_2}^{\upsilon_1} I_{C=O}(\upsilon,D)d\upsilon}{t(D)}$, where $I_{C=O}$ is the baseline-corrected absorbance in the C=O region and t(D) is the thickness remaining after a UV dose D (J cm^{-2}). The chemical degradation rate is evaluated between 0 and 56 J cm^{-2} as $K_{chem} = \frac{A(56)-A(0)}{56}$. The chemical protection factor is $CPF = \frac{K_{chempristine}}{K_{chemcoated}}$.

CPF>1 indicate suppression of photo-chemical bond scission relative to pristine PLA, while CPF<1 indicates poorer protection. All integrations used identical windows and baseline procedures across samples.

Table S2. Normalized carbonyl area A(D) at 0 and 56 J cm⁻², the dose-normalized chemical degradation rate, K_{chem} and the chemical protection factor CPF

	A (D = 0 J cm⁻²)	A (D = 56 J cm⁻²)	K_{chem}	Chemical protection factor
**Pristine PLA**	0.0346	0.371	0.00601	1.00
**10 VPI**	0.0476	0.161	0.00202	2.97
**500 ALD**	0.0524	0.692	0.0114	0.53
**10 VPI + 500 ALD**	0.0275	0.0286	2.0×10^{-5}	3.25×10^{2}

3. The optical properties of ZnO and ZnO-coated PLA films

Upon exposure to UV radiation, ZnO absorbs photons with energy exceeding its band gap, resulting in the excitation of electrons from the valence band to the conduction band and the formation of electron-hole pairs. These pairs are inherently unstable and tend to recombine, a process that releases energy as infrared (IR) radiation or heat. This energy dissipation mechanism reduces the amount of high-energy UV radiation reaching the underlying substrate, thereby ZnO – ALD layers have the potential to protect materials like PLA from photodegradation. The efficient absorption and conversion of UV energy by ZnO are crucial for its role as an effective UV-protective layer. To evaluate the ZnO-ALD layers ability to protect against UV radiation, we first deposited an ~55 nm ZnO-ALD layer on a quartz substrate and measured its optical properties. **Figure S1.** presents the transmittance, reflectance, and absorbance percentages of the ZnO-ALD layer. To isolate the effects of the ZnO-ALD layer, the spectrum of the quartz substrate was subtracted from the sample spectra.

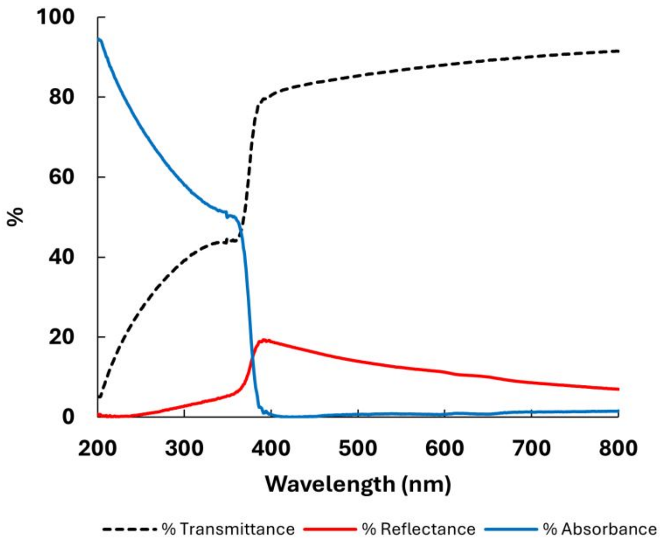

Figure S1. UV-vis spectra of a ~50 nm ZnO - ALD film.

The wide band gap of ZnO, 3.37 eV, corresponding to an absorption edge of 376 nm, allows ZnO to efficiently absorb UV light across a wide range of the UV spectrum, including the critical UV-A (320–400 nm) and UV-B (280–320 nm) regions, thereby preventing harmful

radiation from penetrating the underlying substrate. Unlike other materials such as TiO_2, which do not cover the lower, more energetic UV range (280-320), or CeO_2, which imparts a yellowish tint, ZnO offers excellent UV absorptivity with minimal effect on visible light transparency. At the nanoscale, quantum confinement can further enhance the band gap, shifting absorption to even shorter wavelengths, which improves ZnO's ability to absorb high-energy UV-C (200–280 nm) radiation.[11]

We illustrated the relative contributions of transmittance, reflectance, and absorbance for various models at two specific wavelengths: 254 nm, representing the UV range, and 550 nm, representing visible light, results presented in **Figure S2.** The results reveal that in the UV range, the ZnO-coated PLA (PLA-VPI+ALD) model is predominantly characterized by absorption, demonstrating the ZnO layer's effectiveness in mitigating UV radiation. In contrast, the ZnO layer induces minimal changes to the optical properties in the visible range. These findings indicate that the ZnO layer provides significant UV protection for PLA while maintaining minimal interference with visible light transmission.

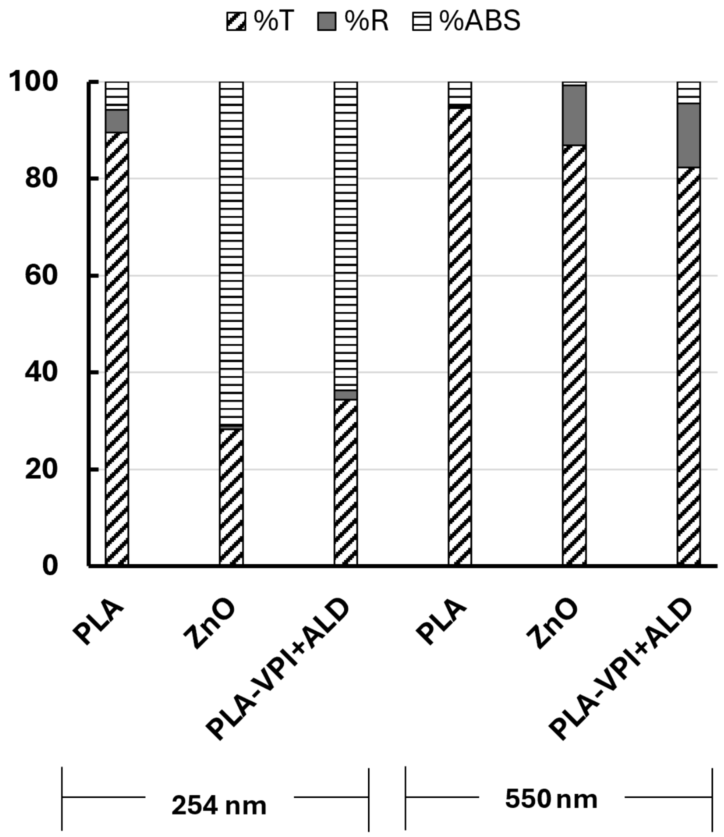

Figure S2. Quantification of the relative contributions of transmittance, reflectance, and absorbance across the different models at two specific wavelengths: 254 nm, representative of the UV range, and 550 nm, indicative of visible light.

UV–Vis spectra were further analysed to quantify the optical blocking properties of the various films at 254 nm and across the UVC band (200–280 nm). To quantify UV blocking,

we first extracted the monochromatic transmittance at 254 nm, T_{254} = T(254), from spectra referenced to a quartz blank. We then computed the optical density at this wavelength, defined as $OD_{254} = -\log_{10}(T_{254})$. Next, assuming Beer–Lambert behavior with film/stack thickness t, we obtained the effective absorption coefficient $\alpha_{eff}(254) = \frac{-\ln(T_{254})}{t}$ and the associated attenuation length $L = \frac{1}{\alpha_{eff}(254)}$. Finally, we quantified broadband performance by calculating the band-averaged transmittance over the UVC range, using an unweighted average $\langle T \rangle_{200-280} = \frac{\int_{200}^{280} T(\lambda) d\lambda}{280-200}$ and reported the band-integrated UV-blocking efficiency as $UBE_{200-280} = 1 - \langle T \rangle_{200-280}$. For pristine PLA, the transmittance at 254 nm was high ($T_{254} \approx 0.90$), corresponding to a very low optical density ($OD_{254} \approx 0.05$) and band-integrated blocking efficiency of only ~12%. These values confirm that pristine PLA is essentially transparent to UVC photons and provides negligible inherent shielding. Although thin PLA films show high transmittance at 254 nm (~90%), even limited absorption is sufficient to induce degradation because each photon carries ~4.9 eV, exceeding the bond dissociation energies of C–C (≈3.6 eV) and C–O (≈3.7 eV) bonds[6]. In the UVO environment, this primary scission is further amplified by ozone and atomic oxygen radicals, leading to rapid erosion of nanometric films despite their apparent optical transparency. By contrast, bulk PLA on the millimeter scale exhibits strong attenuation due to the longer optical path length.

In contrast, ZnO exhibited strong UV attenuation, with $T_{254} \approx 0.28$, $OD_{254} \approx 0.55$, and UBE ≈ 78%. The corresponding effective absorption coefficient was an order of magnitude higher than that of PLA, yielding an attenuation length of ~40 nm compared to ~900 nm for pristine PLA. This analysis highlights the intrinsic capability of ZnO to absorb and block UV radiation and the large contrast between the poor UV resistance of PLA and the excellent UV-blocking performance of ZnO.

Intermediate cases (VPI-only, ALD-only, and VPI+ALD hybrid films) fell between these extremes. Notably, the combined VPI+ALD process improved UV blocking substantially (UBE ≈ 71%), approaching the performance of pure ZnO while maintaining polymer flexibility and transparency in the visible.

Table S3. Summary of T_{254}, OD_{254}, α_{eff}, L and $UBE_{200-280}$

	T_{254}	$OD(T_{254})$	α_{eff}	L (nm)	UBE (200-280nm)
**Pristine PLA**	0.895	0.048	0.0011	902.29	11.72
**10 VPI**	0.873	0.059	0.0014	737.46	14.33
**500 ALD**	0.894	0.049	0.0011	894.18	11.81
**10 VPI + 500 ALD**	0.344	0.463	0.0071	140.56	71.25
**ZnO**	0.282	0.549	0.0253	39.55	77.81

To evaluate the optical properties of the ZnO coating produced via the combined 10 VPI + 500 ALD process, the treatment was applied to a commercial transparent PLA film with a

thickness of 250 μm (**Figure S3A**). Compared to the pristine PLA (**Figure S3B**), the coated film exhibits a slight yellowish tint, indicative of the presence of the ZnO layer. However, the overall transparency of the film is maintained, as evidenced by the clear visibility of the Technion logo positioned behind the sample.

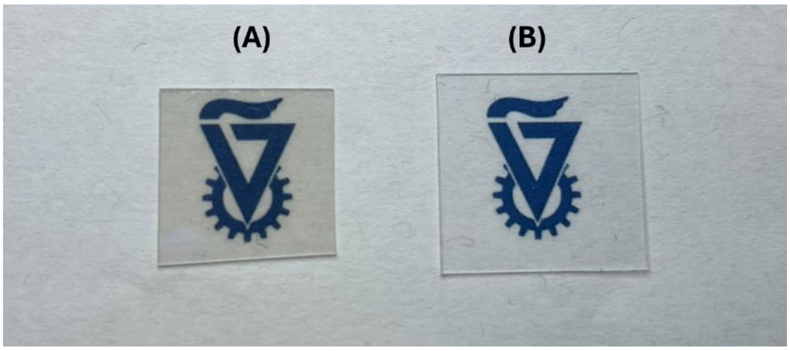

Figure S3. Visual representation of a commercial PLA film that underwent the combined process of 10 cycles of VPI followed by 500 cycles of ALD (A), compared to pristine PLA (B).

4. VPI-derived ZnO nuclei that seed ALD

To contextualize the QCM results, we include a schematic illustration figure (Figure S4) summarizing how VPI cycles generate ZnO_x clusters and particles within polymers and how these structures evolve with cycle number. Prior work by Weisbord *et al.* has shown that ZnO_x crystalline structures appear from the first VPI cycle, and that both particle size and areal density increase with additional cycles, with crystallinity developing as cycles progress. Water retained in the polymer is a key driver of this evolution.[12] These ZnO_x particles are embedded within the polymer but also develop on the polymer's surface, and thus act as nucleation sites for the first few ALD cycles, enabling rapid transition to steady ALD growth once a continuous surface forms.

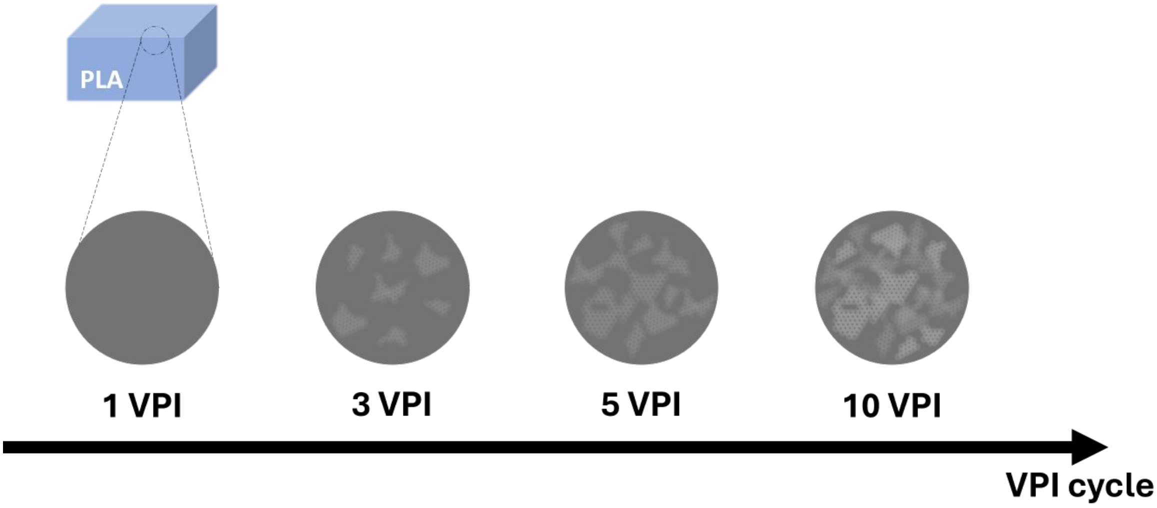

Figure S4. Schematic illustration of the evolution of VPI-grown ZnO_x nuclei with cycle number. Increasing VPI cycles (e.g., 1, 3, 5) illustrate the transition from dispersed sub-nm clusters to embedded polycrystalline nanoparticles, with a rise in both particle size and density as cycles advance. This illustrates how VPI creates ZnO_x clusters and particles on and within PLA that seed ZnO-ALD from the initial cycles.

5. XPS

XPS corroborates VPI-enabled subsurface Zn species that seed ALD. Surveys show Zn appears after VPI, increases after VPI+ALD, and remains after etch (main text, Figure 5A and Figure 5D). The Zn chemical state was assigned from Zn 2p and Zn LMM via the modified Auger parameter (values given in the main text), indicating hydroxylated Zn at the near surface. Supporting core levels are provided here: O 1s (Figure S4) is dominated by PLA/–OH at ~532–533 eV with no distinct ~530 eV lattice-O component, consistent with the α′ assignment; C 1s (Figure S5) retains PLA ester features and provides the 284.8 eV charge reference. Table S4 lists area-based atomic % (C, O, Zn) for all samples; the non-zero Zn fraction after etch evidences subsurface Zn that persists beneath the removed top layer.

Table S4. XPS elemental composition (atomic %) of C, O, and Zn for (a) PLA, (b) PLA + VPI, (c) PLA + VPI + ALD, and (d) PLA + VPI + ALD after etch.

sample	%C	%O	%Zn
**PLA**	61.6	38.4	0.0
**PLA VPI**	59.2	38.1	2.7
**PLA VPI+ALD**	58.0	37.3	4.8
**PLA VPI+ALD after etch**	62.5	36.8	0.7

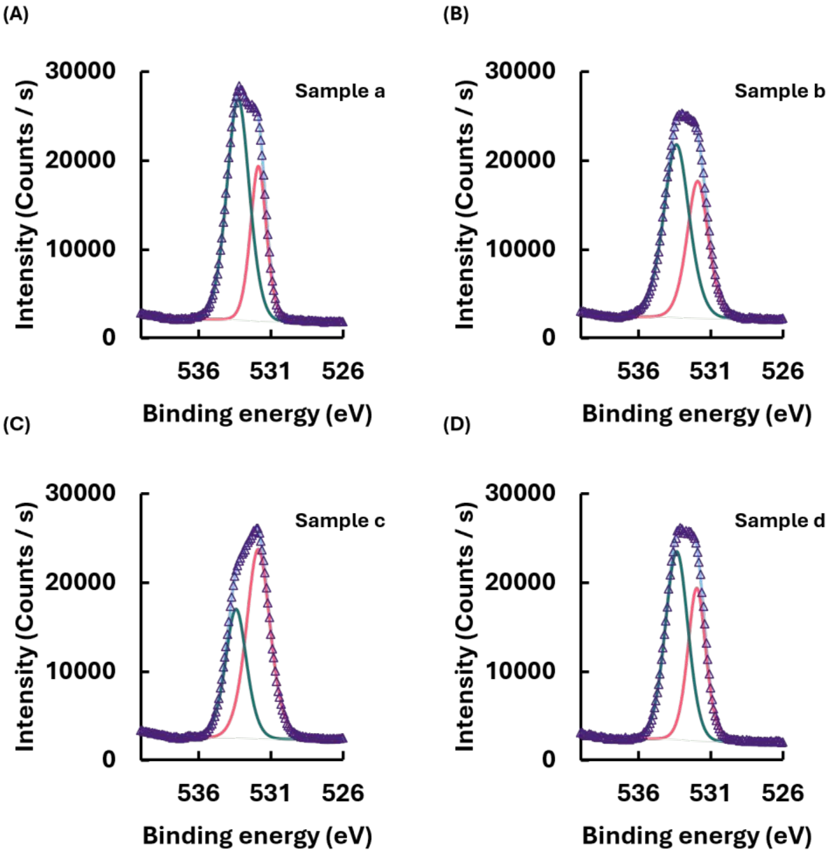

Figure S5. High-resolution O 1s spectra show that all samples are dominated by the PLA/–OH envelope near ~532–533 eV; no additional ~530 eV lattice-oxygen peak is required, consistent with hydroxylated Zn at the near surface rather than bulk ZnO. Spectra are shown for: (a) PLA, (b) PLA + VPI, (c) PLA + VPI + ALD, and (d) PLA + VPI + ALD after etch.

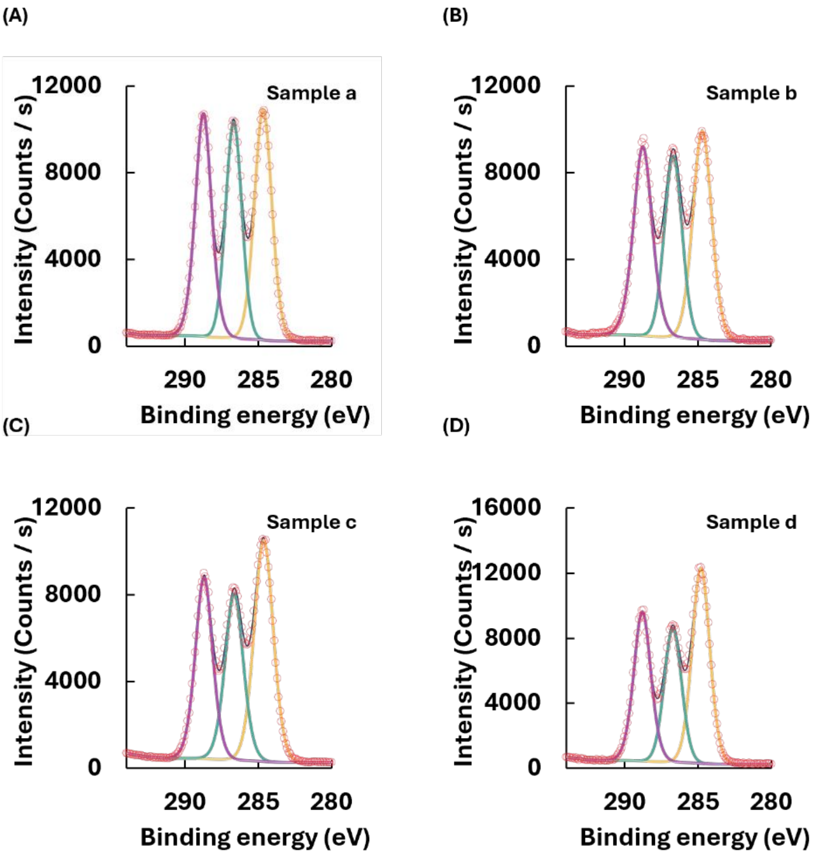

Figure S6. High-resolution C 1s spectra, (a) PLA, (b) PLA + VPI, (c) PLA + VPI + ALD, and (d) PLA + VPI + ALD after etch.

6. PLA thin films thickness measurements

Table S5 presents thickness measurements obtained using an ellipsometer (Alpha-SE, J.A. Woollam Co. Inc.) for PLA samples subjected to various ALD processes. The table includes data on different substrates, specifically thin PLA (~100nm) and thick PLA films (~400nm), as well as a silicon wafer. For each sample, the table details the ALD process applied and the corresponding ZnO layer thickness.

Table S5. Ellipsometry thickness measurements for ALD processes using Alpha-SE.

Substrate	Recipe	ZnO thickness (nm)
Thin PLA	500 ALD	1.7 ± 1.1
Thick PLA	500 ALD	3.1 ± 0.1
Si	500 ALD	56.4 ± 0.7
Thin PLA	10 VPI + 500 ALD	52.8 ± 3.0
Thick PLA	10 VPI + 500 ALD	56.8 ± 0.6
Si	10 VPI + 500 ALD	57.6 ± 0.4

Figure S7 presents the thickness measurement of a ZnO layer (~55nm) deposited on a thick PLA film subjected to a combined process of 10 VPI cycles followed by 500 ALD cycles. The measurement was conducted using scanning electron microscopy (SEM) after sample preparation via focused ion beam (FIB) milling for transmission electron microscopy (TEM) analysis.

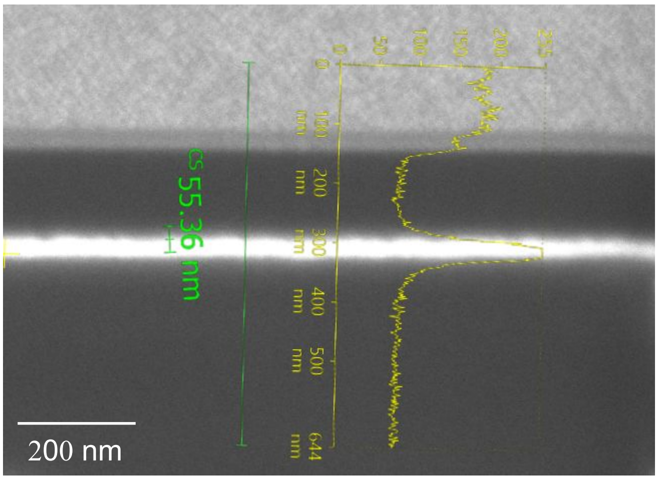

Figure S7. FIB-SEM cross section images of PLA thick film after the combined process of 10 VPI cycles followed by 500 ALD cycles - the measured ZnO layer thickness is ~55 nm.

References

(1) Andrady, A. L. Ultraviolet Radiation and Polymers. In *Physical Properties of Polymers Handbook*; Mark, J. E., Ed.; Springer: New York, NY, 2007; pp 857–866. https://doi.org/10.1007/978-0-387-69002-5_51.

(2) GUILLET, J. Fundamental Processes in the Photodegradation of Polyolefins. In *Stabilization and Degradation of Polymers*; Advances in Chemistry; AMERICAN CHEMICAL SOCIETY, 1978; Vol. 169, pp 1–10. https://doi.org/10.1021/ba-1978-0169.ch001.

(3) Geuskens, G. Chapter 3 Photodegradation of Polymers. In *Comprehensive Chemical Kinetics*; Bamford, C. H., Tipper, C. F. H., Eds.; Degradation of Polymers; Elsevier, 1975; Vol. 14, pp 333–424. https://doi.org/10.1016/S0069-8040(08)70335-2.

(4) Carlsson, D. J.; Wiles, D. M. The Photodegradation of Polypropylene Films. II. Photolysis of Ketonic Oxidation Products. *Macromolecules* **1969**, *2* (6), 587–597. https://doi.org/10.1021/ma60012a006.

(5) Torikai, A.; Ohno, M.; Fueki, K. Photodegradation of Poly(Methyl Methacrylate) by Monochromatic Light: Quantum Yield, Effect of Wavelengths, and Light Intensity. *J. Appl. Polym. Sci.* **1990**, *41* (5–6), 1023–1032. https://doi.org/10.1002/app.1990.070410513.

(6) Rabek, Jan. F. Physical Aspects of the Photodegradation of Polymers. In *Polymer Photodegradation: Mechanisms and experimental methods*; Rabek, Jan. F., Ed.; Springer Netherlands: Dordrecht, 1995; pp 1–23. https://doi.org/10.1007/978-94-011-1274-1_1.

(7) Guillet, J. Photochemistry and Molecular Motion in Solid Amorphous Polymers. In *Advances in Photochemistry*; John Wiley & Sons, Ltd, 1988; pp 91–133. https://doi.org/10.1002/9780470133446.ch2.

(8) Mark, H. F. *Encyclopedia of Polymer Science and Technology, Concise*; John Wiley & Sons, 2013.

(9) Belbachir, S.; Zaïri, F.; Ayoub, G.; Maschke, U.; Naït-Abdelaziz, M.; Gloaguen, J. M.; Benguediab, M.; Lefebvre, J. M. Modelling of Photodegradation Effect on Elastic–Viscoplastic Behaviour of Amorphous Polylactic Acid Films. *J. Mech. Phys. Solids* **2010**, *58* (2), 241–255. https://doi.org/10.1016/j.jmps.2009.10.003.

(10) Zaaba, N. F.; Jaafar, M. A Review on Degradation Mechanisms of Polylactic Acid: Hydrolytic, Photodegradative, Microbial, and Enzymatic Degradation. *Polym. Eng. Sci.* **2020**, *60* (9), 2061–2075. https://doi.org/10.1002/pen.25511.

(11) Lin, K.-F.; Cheng, H.-M.; Hsu, H.-C.; Lin, L.-J.; Hsieh, W.-F. Band Gap Variation of Size-Controlled ZnO Quantum Dots Synthesized by Sol–Gel Method. *Chem. Phys. Lett.* **2005**, *409* (4), 208–211. https://doi.org/10.1016/j.cplett.2005.05.027.

(12) Weisbord, I.; Barzilay, M.; Cai, R.; Welter, E.; Kuzmin, A.; Anspoks, A.; Segal-Peretz, T. The Development and Atomic Structure of Zinc Oxide Crystals Grown within Polymers from Vapor Phase Precursors. *ACS Nano* **2024**, *18* (28), 18393–18404. https://doi.org/10.1021/acsnano.4c02846.
